# Adalimumab Treatment Effects on Inflammation and Adipose Tissue Mitochondrial Respiration in Hidradenitis Suppurativa

**DOI:** 10.1002/edm2.70002

**Published:** 2024-10-02

**Authors:** Ronni Eg Sahl, Axel Illeris Poggi, Valdemar Wendelboe Nielsen, Yiqiu Yao, Ioanna Patsi, Steen Seier Poulsen, Flemming Dela, Steen Larsen, Simon Francis Thomsen, Jørn Wulff Helge

**Affiliations:** ^1^ Xlab, Center for Healthy Aging, Department of Biomedical Sciences, Faculty of Health and Medical Sciences University of Copenhagen Copenhagen Denmark; ^2^ Department of Dermatology Bispebjerg‐Frederiksberg University Hospital Copenhagen Denmark; ^3^ Department of Biomedical Sciences, Faculty of Health and Medical Sciences University of Copenhagen Copenhagen Denmark; ^4^ Department of Geriatrics Bispebjerg‐Frederiksberg University Hospital Copenhagen Denmark; ^5^ Clinical Research Centre Medical University of Bialystok Bialystok Poland; ^6^ Department of Institute of Sports Medicine Copenhagen, Department of Orthopedic Surgery M Bispebjerg‐Frederiksberg University Hospital Copenhagen Denmark

**Keywords:** adalimumab, adipose tissue, hidradenitis suppurativa, inflammation, low‐grade inflammation, macrophages, mitochondrial respiration

## Abstract

**Objective:**

Tumour necrosis factor (TNF)‐α is a proinflammatory marker and has been shown to affect mitochondrial function in different tissues. We investigated the effect on adipose tissue (AT) inflammation and mitochondrial respiration in patients with hidradenitis suppurativa (HS) after 12 weeks of treatment with adalimumab, a TNF‐α inhibitor.

**Methods:**

We sampled blood and an AT biopsy from 13 patients with HS and 10 control subjects after an overnight fast. The patients were retested after at least 12 weeks of treatment with adalimumab (40 mg/week). We measured macrophage content and mitochondrial respiration in the AT and interleukin (IL)‐1β, IL‐6, IL‐10, high‐sensitivity C‐reactive protein (hsCRP), interferon‐γ, TNF‐α, adiponectin and leptin in plasma. Clinical scores and Dermatology Quality of Life Index (DLQI) were assessed.

**Results:**

We found a higher anti‐inflammatory macrophage content (CD206^+^) in the patient group compared with the control group, but no differences between before and after the intervention. No difference in mitochondrial respiration was observed. We observed higher plasma IL‐6 and hsCRP concentrations in patients with HS compared to controls, with no differences before and after the intervention. The difference between controls and HS patients was abolished after the intervention. HS patients improved their DLQI after the intervention with no change in clinical scores.

**Conclusion:**

Treatment with adalimumab in patients with HS does not alter AT inflammation or mitochondrial respiratory capacity; however, we did see a higher content of anti‐inflammatory macrophages in the patient group compared with the control group.

## Graphical abstract

Adalimumab led to improvements in self‐reported quality of life but did not yield statistically significant changes in clinical scores. A key finding was that mitochondrial respiration in HS patients was similar to healthy controls and was not altered by the treatment. The macrophage profile in adipose tissue exhibited more anti‐inflammatory characteristics than in healthy controls; however, this did not relate to systemic low‐grade inflammation, and adalimumab had no impact on these measures.

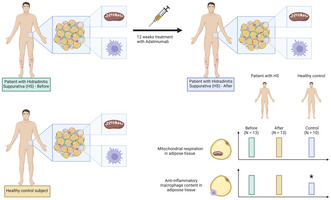

## 1 Introduction

Hidradenitis suppurativa (HS) is a chronic inflammatory skin disease characterised by the formation of inflammatory nodules, abscesses and sinus tracts in intertriginous regions of the body. The prevalence is reported to be between 0.1%–4%, and there is an overrepresentation of the disease among women, individuals with obesity and smokers [1, 2, 3, 4]. The disease has a negative impact on quality of life [5] and is associated with an increased risk of mental illness [6, 7] and cardiovascular disease [8, 9]. Treatment involves topical ointments, antibiotics and in severe cases surgery and biological treatment. Adalimumab, a monoclonal antibody targeting TNF‐α was the first licensed biological treatment for HS, and weekly treatment significantly decreased inflammatory lesion count in HS patients [10] and improved quality of life [11]. TNF‐α, IL‐1β and IL‐10 are increased many‐fold in lesional HS skin; therefore, treatment targeting these specific cytokines is a favourable strategy [12]. The use of anti‐TNF‐α decreases the bioavailability of TNF‐α both systemically and in the affected skin areas. Whether systemic inflammation is a contributing factor in the development of the disease is not fully understood. Complement activation has been speculated to contribute to the activation of HS [13], but it has not been shown that systemic inflammation is important in the activation of HS. In lesional HS skin, innate immune cells are highly abundant, and M1 macrophages are upregulated while M2 macrophages are downregulated [14]. M1 macrophages release pro‐inflammatory cytokines with local and systemic effects as these markers are released into the circulation. In a related skin disease, psoriasis, increased M1 polarisation is observed [15], which correlates with disease severity. In the same study, treatment with adalimumab decreased M1 polarisation. Since HS and psoriasis share certain pathogenic mechanisms, similar effects could be expected in HS patients. Congruently, Mariottoni and colleagues speculated that pro‐inflammatory macrophages could play a central role in HS pathogenesis and could also serve as HS biomarkers and therapeutic targets [16], underlining the importance of macrophage content and polarisation in HS.

Inflammatory markers can affect mitochondrial function negatively. In cardiomyocytes, high concentrations of TNF‐α or IL‐1α decreased oxygen consumption through the mitochondrial respiratory chain complexes [17]. Interestingly, this effect disappeared with anti‐TNF‐α treatment, indicating a direct effect of TNF‐α on mitochondria in cardiomyocytes. If mitochondria in adipose tissue (AT) have a similar response to TNF‐α, we speculate that mitochondrial respiration is inhibited in AT in HS patients and treatment with anti‐TNF‐α could increase mitochondrial respiration, by decreasing local and systemic TNF‐α levels. To our knowledge, whether a change in systemic inflammation can affect the inflammation profile and mitochondrial function in AT has not yet been investigated.

Changes in body fat mass have been demonstrated to influence macrophage infiltration and polarisation [18, 19, 20, 21, 22]. Macrophages in AT release inflammatory markers promoting systemic low‐grade inflammation. Whether alterations in low‐grade inflammation can work the other way around and affect AT is not described. Consequently, we hypothesise that patients initiating adalimumab treatment may serve as a model for investigating how shifts in systemic low‐grade inflammation can influence inflammation within AT. The current study aimed to investigate whether treatment with adalimumab in HS patients decreases systemic inflammation and whether this positively affects AT inflammation and mitochondrial respiration.

## 2 Methods

### 2.1 Participants and Ethical Approval

The study includes a patient group with HS and a healthy control group. The patient group was recruited when treatment of HS with adalimumab was initiated at the Department of Dermatology at Bispebjerg Hospital, Copenhagen. Patients underwent regular clinical assessment in the outpatient clinic before enrolment in the study. This included assessment of pain via the visual analogue scale (VAS), disease severity via International Hidradenitis Suppurativa Severity Score System (IHS4) and quality of life via Dermatology Life Quality Index (DLQI). Disease severity was divided into three stages: Hurley Stages I, II and III. This index is not dynamic and was not measured after the treatment period.

The control group was recruited with advertisement through a bureau (forskningnu.dk). Written informed consent was obtained for all subjects. The study was performed in accordance with the Declaration of Helsinki and was approved by the local ethics committee in Copenhagen (H‐18057641).

**FIGURE 1:**
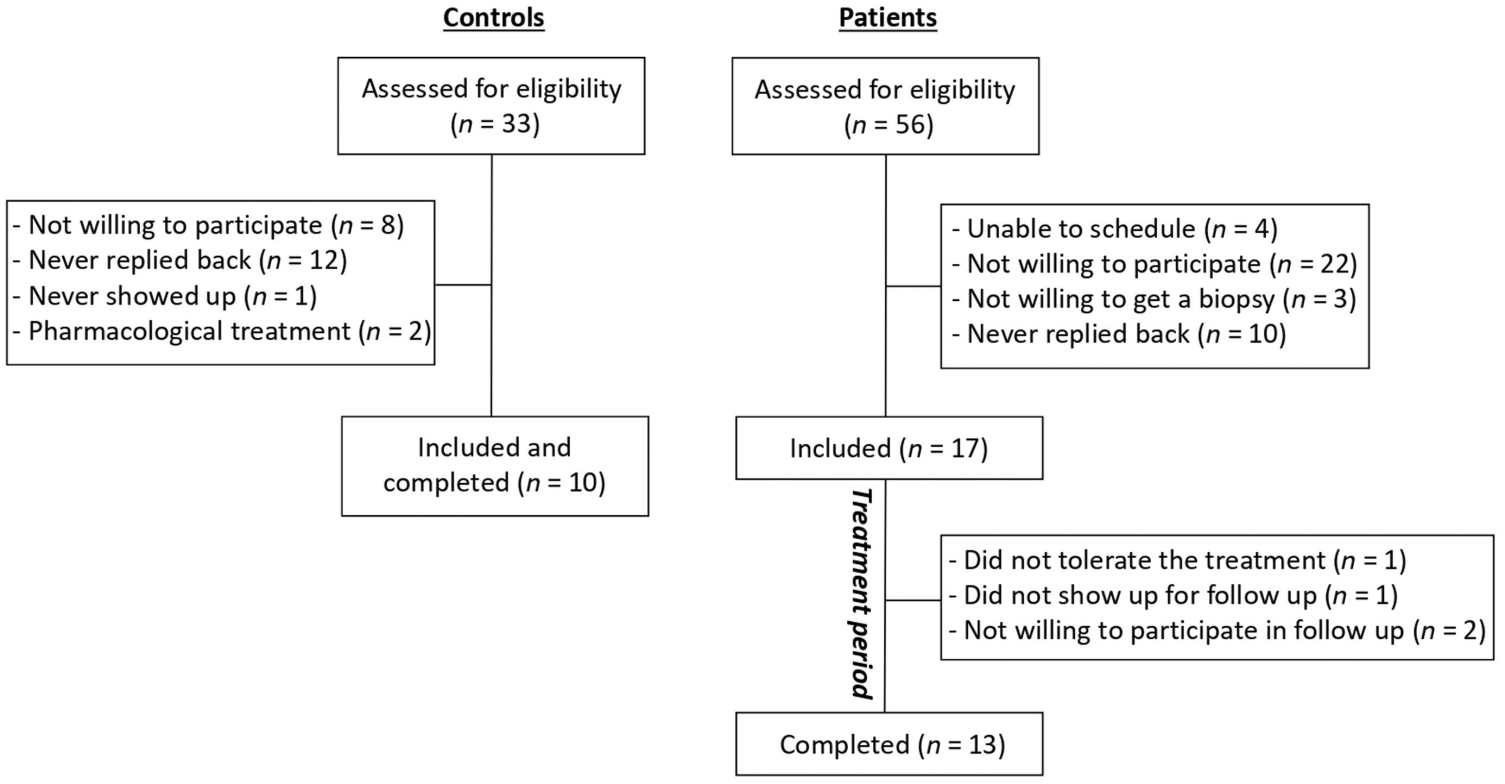
Flow chart of test subjects.

Patients were recruited between March 2019 and November 2022. Control subjects were recruited between October 2022 and January 2023. In total, 13 patients completed the intervention period and follow‐up test, and 10 control subjects completed the test day (see Figure 1 for a flow chart of recruiting).

### 2.2 Study Design

Hidradenitis suppurativa (HS) patients and control subjects came in after an overnight fast. They filled out the DLQI questionnaire. Height and weight were measured, and blood was sampled from vena mediana cubiti. Lastly, a biopsy was obtained from the subcutaneous abdominal depot close to the umbilicus. After the biopsy, patients received their first injection with adalimumab (160 mg). They received a second dose (80 mg) after 14 days and then continued with a weekly dose of 40 mg, after additional 14 days, for at least 8 weeks and a max of 12 weeks before the follow‐up. The follow‐up test was performed in the same way as the initial test. Control subjects were only tested once. As not all patients were examined on clinical parameters in the outpatient clinic after the 12 weeks of treatment, the data are presented for a subset of the patient group. DLQI was measured twice in the patient group before start‐up on treatment. We have used the data obtained on the test day, not the one obtained in the outpatient clinic.

### 2.3 Biopsy Procedure

A biopsy was obtained under local anaesthesia and through a 10 mm incision in the skin, using the modified Bergstrom procedure with suction. The biopsy was obtained from a non‐affected area of the skin. The biopsy was quickly dissected free of blood and connective tissue using forceps and divided into three portions. One portion was placed in ice‐cold fixative (Zamboni formaldehyde [4%]), one portion was placed in ice‐cold BIOPS buffer (see specifications below) and one portion was snap‐frozen in liquid nitrogen.

### 2.4 Mitochondrial Respiration

The adipose tissue was kept and dissected in ice‐cold BIOPS buffer (final concentration: CaK_2_EGTA [2.77 mM], K_2_EGTA [7.23 mM], Na_2_ATP [5.77 mM], MgCl_2_6 H_2_O [6.56 mM], taurine [20 mM], Na_2_phospho‐creatine [15 mM], imidazole [20 mM], dithiothreitol [0.5 mM], MES [50 mM], pH = 7.1, 0°C), using forceps and a magnifier. After thorough dissection, the adipose tissue was washed for 10 min in ice‐cold MIR05 buffer (final concentrations: sucrose [110 mM], potassium lactobionate [60 mM], EGTA [0.5 mM], MgCl_2_6 H_2_O [3 mM], taurine [20 mM], KH_2_PO_4_ [10 mM], HEPES [20 mM], BSA [1 g/L], pH 7.1, 37°C). After wash, MIR05 was gently removed using filter paper, and the AT was weighed out in portions of approximately 20 mg per chamber. Mitochondrial respiration was performed in duplicates using high‐resolution respirometry (Oxygraph‐2 K, Oroboros instruments, Innsbruck, Austria). We used the following protocol (final concentrations): Digitonin (2 μM) to permeabilise the plasma membrane of the adipocyte. Malate (2 mM), pyruvate (5 mM) and glutamate (10 mM) to measure leak respiration. ADP was added (12.5 mM) to Measure State three respiration (complex I stimulated respiration; CI_*P*_). Succinate was added step‐wise (0.1—48 mM) to measure succinate sensitivity and Maximal State three respiration (complex I + II stimulated respiration, CI + II_*P*_). Cytochrome *c* (10 μM) was added to test the integrity of the outer mitochondrial membrane. Oligomycin (2 μg/mL) was added to inhibit the ATP synthase (state 4o) and finally carbonyl cyanide‐4‐(trifluoromethoxy) phenylhydrazone (FCCP) was titrated in steps of 0.5 μM to test the capacity of the electron transport system (ETS).

Mitochondrial respiration analysis performed in the study was done in fresh tissue. The samples were transported from Bispebjerg Hospital to the Panum Institute, University of Copenhagen and the analyses were therefore delayed by about 60–120 min from sampling to the start of dissection. Since we could not circumvent the delay, we previously measured the effect of delaying the analyses for 3 h and found that it did not affect the measurements [23].

### 2.5 Macrophage Content

The AT was kept in Zamboni fixative for 24 h and then in 70% alcohol until paraffin embedding. Sections of 6 μm were cut on a microtome (Epredia HM355s, Michigan, USA) and mounted on a microscopic slide. Samples were stained immunohistochemically with macrophage‐specific primary antibodies (CD14 [mouse, Santa Cruz, Texas, USA], CD163 [mouse, Novocastra, Germany] and CD206 [rabbit, Cell Signaling, Massachusetts, USA]) overnight at 4°C and then washed with PBS (Phosphate Buffered Saline), stained with secondary antibody to amplify the reaction and washed again in PBS, then incubated with DAB (3,3′‐diaminobenzidine) and Mayer's haematoxylin (Sigma‐Aldrich, Denmark) to counterstain for better visualisation and contrast. Lastly a final wash with PBS.

The sections were quantified under a Zeiss Axioskop 2 Plus microscope (Carl Zeiss, Germany) using a 10× objective. Two representative areas of the biopsy were saved using a Deltapix camera (Deltapix, Denmark). Each area was quantified twice and the average of the four quantifications of each biopsy was used as the final value.

### 2.6 Blood Samples

Blood samples were spun down at 2772G for 10 min at 4°C to separate red blood cells and plasma. Plasma was stored at −80°C until analyses. We used an inflammatory multiplex assay from Meso Scale (Maryland, USA) to measure interleukin (IL)‐6, IL‐10, IL‐1β, tumour necrosis factor α (TNF‐α), Interferon (IFN)‐γ, plasma concentrations. We measured Adiponectin plasma concentrations with an RIA kit (Sigma‐Aldrich, Missouri, USA) and Leptin plasma concentrations using an ELISA kit (R&D Systems, Minneapolis, USA). High‐sensitivity *C*‐reactive protein (hsCRP) plasma concentrations were measured with Cobas (Roche, Germany).

### 2.7 Statistical Analyses

A paired *t*‐test was performed on before and after the intervention data from the patient group. An unpaired *t*‐test between the patient group (before data) and the control group was performed and likewise an unpaired *t*‐test between the patient group (after data) and the control group. Succinate sensitivity (*K*_m_) was calculated as ½ maximal velocity (*V*_max_) using Michaelis Menten kinetics. The smoking status was analysed with a non‐parametric Mann–Whitney test. The number of samples included differs between analyses due to missing samples and outliers. All analyses and graphs are performed in GraphPad Prism 9.5 (GraphPad Software, California, USA).

## 3 Results

### 3.1 Anthropometrics and Clinical Scores

The HS patient group and control group were matched on age, sex, height and weight.

No change in body weight was seen in the HS patient group after the intervention (Table 1). Quality of life was measured with the DLQI and the HS patient group had a higher score than the control group (*p* < 0.001), and the DLQI score decreased in the HS patient group after the intervention (*p* < 0.01) (Table 1). For a subset of the HS patient group, we had the IHS4 (International Hidradenitis Suppurativa severity score system) and pain score. We found no statistically significant changes in these two parameters despite a numerical decrease.

**TABLE 1:** Subject characteristics.

	Control (*n* = 10)	HS patients (*n* = 13)		*p* value	
		Before	After	Control versus before	Before versus after
Sex (F/M)	7/3	9/4		0.97	
Smokers/former smokers/non‐smokers	0/3/7	7/4/2		< 0.01	
Age (years)	40 ± 3	39 ± 4		0.93	
Height (cm)	175 ± 2	174 ± 3		0.76	
Weight (kg)	94.6 ± 4.8	96.2 ± 5.5	98.1 ± 5.5	0.83	0.18
BMI (kg/m^2^)	31.0 ± 1.6	31.8 ± 1.5	32.4 ± 1.5	0.72	0.19
DLQI	0.1 ± 0.1	17.3 ± 2.1	11.4 ± 1.6	< 0.001	< 0.01
Hurley stage (I/II/III)		(1/9/3)			
Neutrophil to lymphocyte ratio (NLR)		3.0 ± 0.3			

		Subset of HS patients			
		Before	After		
IHS4 score (*n* = 10)		12.3 ± 4.2	9.5 ± 3.1		0.41
Pain score (*n* = 8)		7.8 ± 0.5	7.4 ± 0.5		0.43

*Note:* A subset of patients was seen at the outpatient clinic after 12 weeks of adalimumab treatment and clinical scores were obtained.

Abbreviations: BMI, body mass index; DLQI, Dermatology Life Quality Index; HS, hidradenitis suppurativa; IHS4, International Hidradenitis Suppurativa Severity Score System.

### 3.2 Macrophages Content in Adipose Tissue

**FIGURE 2:**
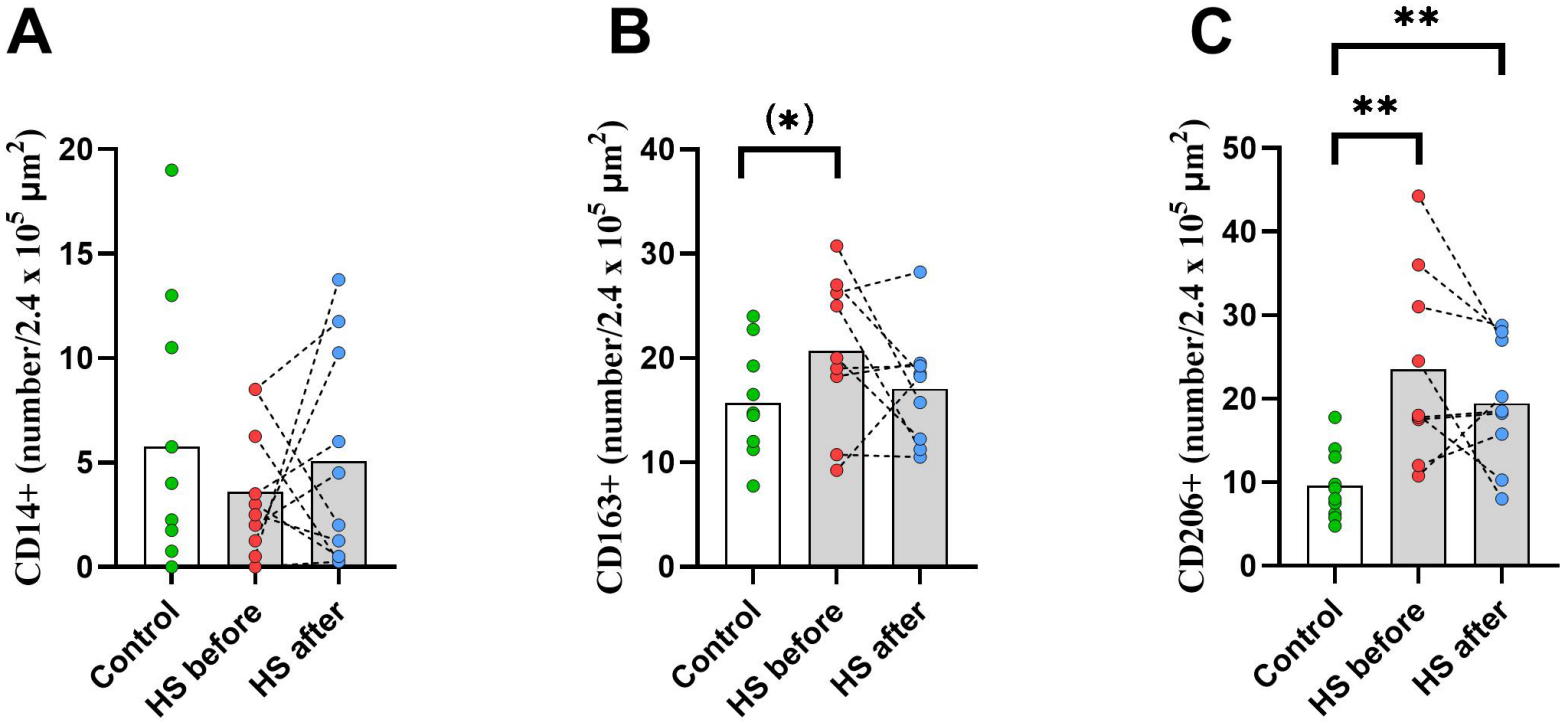
CD14^+^ (A), CD163^+^ (B) and CD206^+^ (C) macrophage content in adipose tissue in controls (white box, green dots) and in the patient group before (grey box, red dots) and after (grey box, blue dots) 12 weeks of adalimumab treatment. *p < 0.10, **p < 0.01. HS, hidradenitis suppurativa.

The number of CD14^+^ macrophages was not different in the control group compared with the HS patient group, both before and after the intervention (*p* = 0.27 and *p* = 0.66, respectively), and no difference in the HS patient group before and after the invention was observed (*p* = 0.47). Figure 2A.

The number of CD163^+^ macrophages did not change before and after the intervention in the HS patient group (*p* = 0.39). Before the intervention, a tendency towards a higher content in the HS patient group compared with the control group was observed (*p* = 0.07). After the intervention, there was no difference between the HS patient group and the control group (*p* = 0.32). Figure 2B.

The number of CD206^+^ macrophages was higher in the HS patient group both before and after the intervention compared with the control group (*p* < 0.01). No difference before and after the intervention was observed in the HS patient group (*p* = 0.56), Figure 2C.

We found a positive correlation between changes in CD14^+^ macrophages and pain score (*r* ^2^ = 0.74, *p* < 0.05, *n* = 6) and a negative correlation between changes in both CD163^+^ (*r* ^2^ = 0.77, *p* < 0.05, *n* = 5) and CD206^+^ (*r* ^2^ = 0.82, *p* < 0.05, *n* = 5) macrophages and pain score, data not shown.

### 3.3 Mitochondrial Respiration in Adipose Tissue

**FIGURE 3:**
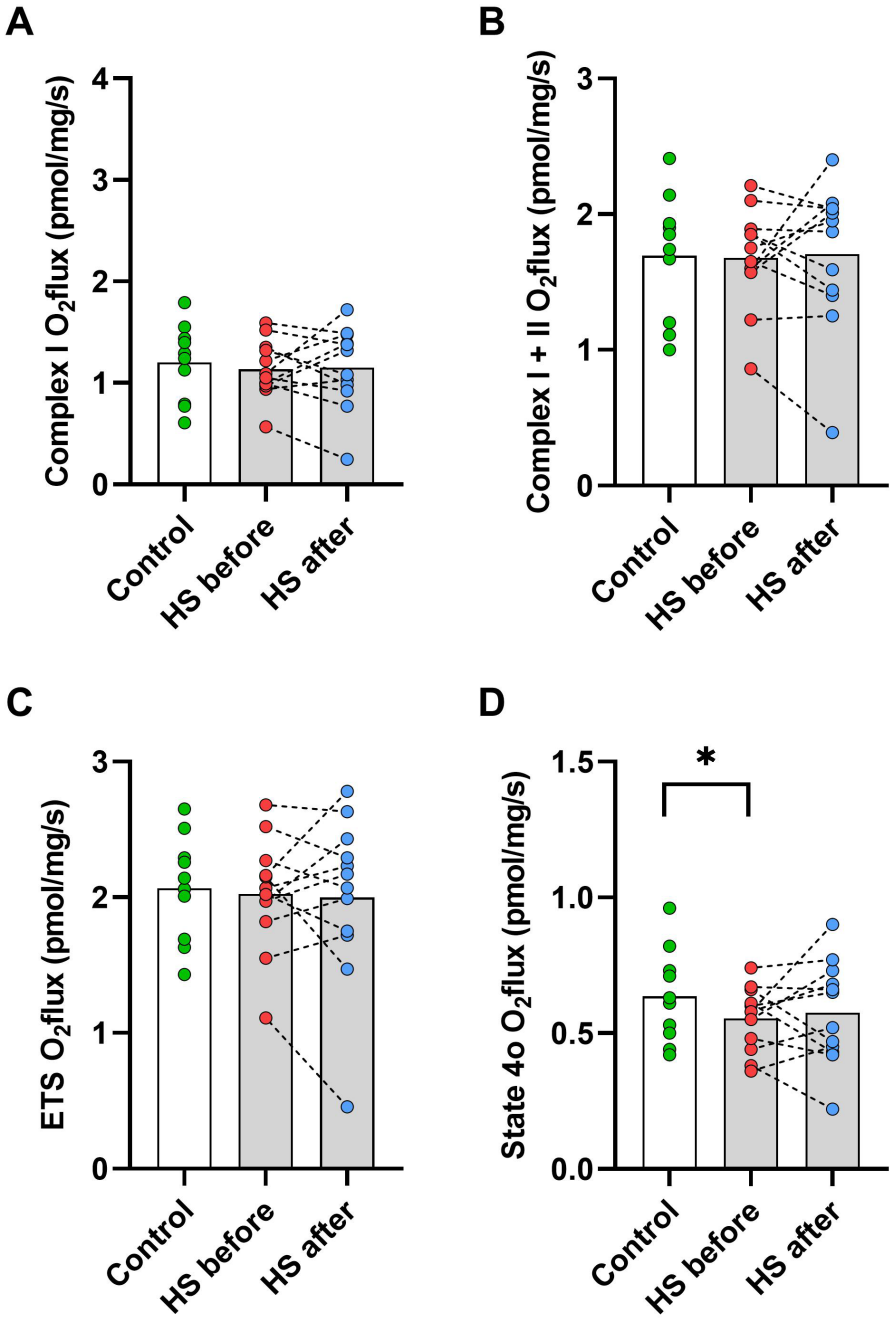
Mitochondrial respiration in adipose tissue shown as O_2_ flux (pmol/mg/s) for complex I (A), complex I + II (B) and the total electron transport system (ETS) (C). Also, respiration after inhibition of the ATP synthase (state 4o) is shown (D). White box and green dots are the control group. Grey boxes are patient group before (red dots) and after (blue dots) 12 weeks of treatment with adalimumab. *p < 0.05. HS, hidradenitis suppurativa.

There were no differences in mitochondrial respiration between the control group and the HS patient group for either Complex I (Figure 3A), Complex I + II (3B) or ETS respiration (3C). There was no effect of the intervention in the patient group. State 4o respiration was higher in the control group than in the HS patient group before the intervention (*p* < 0.05, Figure 3D), but not after the intervention.

Succinate sensitivity, calculated by Michaelis–Menten kinetics using concentrations between 0.1 and 48 mM, showed no differences between either control (*K*_m_: 1.88 mM) and patient group before (*K*_m_: 2.24 mM, *p* = 0.60) or after (*K*_m_: 1.75 mM, *p* = 0.66). Also, no difference between before and after in the patient group was observed (*p* = 0.33).

### 3.4 Biomarkers of Systemic Inflammation

**FIGURE 4:**
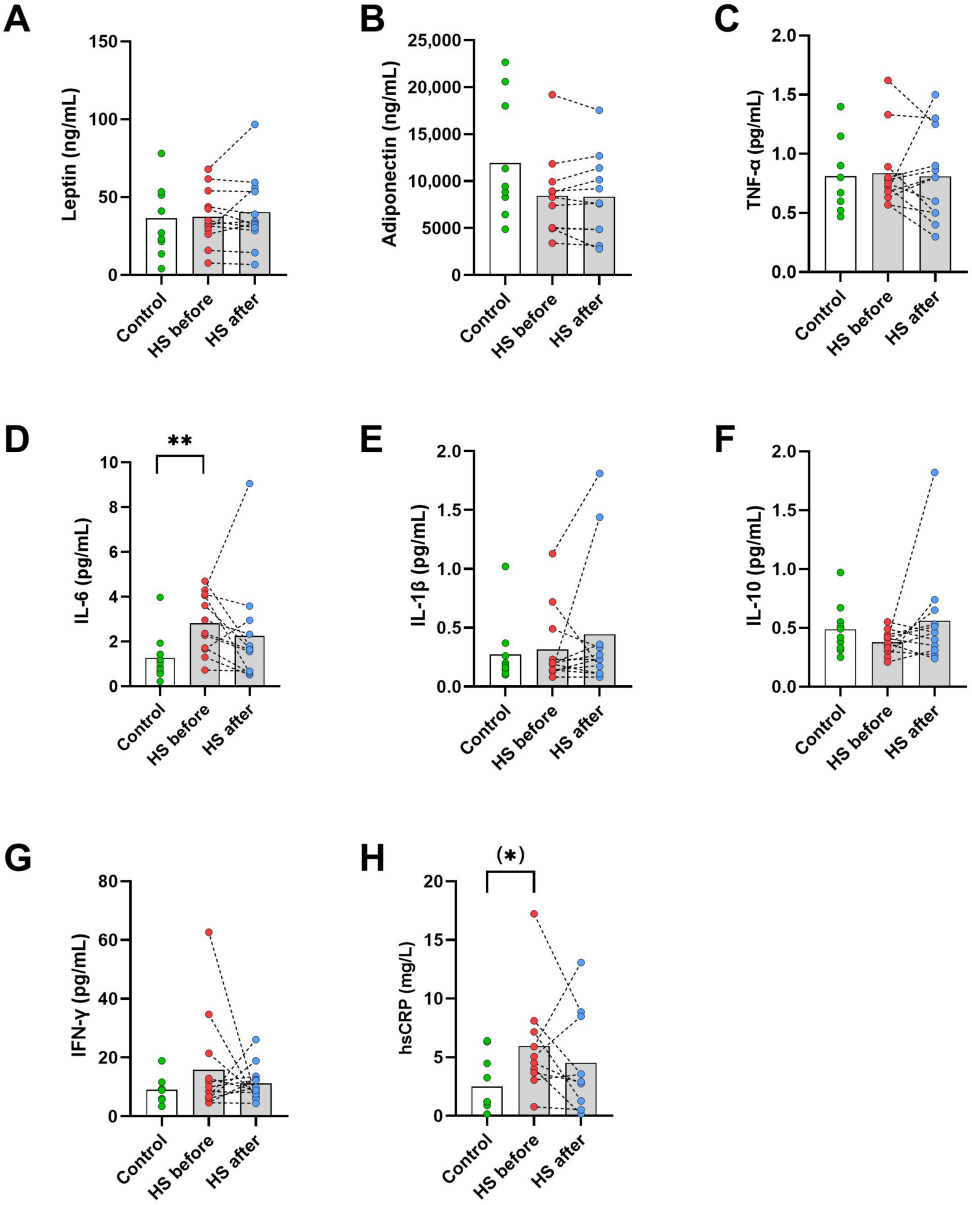
Plasma concentrations of Leptin (A), adiponectin (B), TNF‐α (C), IL‐6 (D), IL‐1β (E), IL‐10 (F), IFN‐γ (G) and hsCRP (H). *p < 0.05, **p < 0.01. White box and green dots are the control group. Grey boxes are patient group before (red dots) and after (blue dots) 12 weeks of treatment with adalimumab. HS, hidradenitis suppurativa.

Plasma concentration of leptin (Figure 4A), adiponectin (4B), TNF‐α (4C), IL‐1β (4E), IL‐10 (4F) and IFN‐γ (4G) was not different between the control group and the HS patient before or after the intervention. Also, no differences between before and after the intervention in the HS patient group were observed. Plasma IL‐6 (4D) concentration was higher in the HS patient group before the intervention than in the control group (*p* < 0.01). No difference between before and after the intervention and between the control group and the HS patient group after the intervention was observed. A tendency towards a higher plasma concentration of hsCRP (4H) in the HS patient group before the intervention and the control group was observed (*p* = 0.06), with no difference between the control and HS patient group after the intervention. Also no difference between the HS patient group before and after was seen.

From the patient group, we had baseline measurements of different blood cells. The neutrophil‐to‐lymphocyte ratio (NLR) was 3.0.

## 4 Discussion

Despite an inflammatory cutaneous manifestation in HS, we observed a more anti‐inflammatory profile in AT in the HS patients than in healthy controls. We found no differences in mitochondrial respiration between the two groups and only a marginally higher systemic inflammation. Treatment with adalimumab did not change any parameters from before to after the intervention in the HS patient group.

We found a higher content of anti‐inflammatory macrophages in the HS patient group compared with the control group. This is somewhat surprising since HS is considered an inflammatory disease where disease‐affected areas of the skin have been shown to express a more proinflammatory profile with higher M1 and lower M2 macrophage content [16], and with pus containing high levels of proinflammatory cytokines [24] A higher proportion of M1 macrophages results in a higher release of proinflammatory cytokines [6] that are taken up in the circulation and contribute to low‐grade inflammation. However, we did not see a pathological inflammation profile in the blood and likewise, the adipose tissue did not have an inflammatory profile. This divergent difference in local inflammatory profile between tissues in HS patients has been shown earlier by Kanni and colleagues where stimulated isolated blood mononuclear cells (PBMC) release less cytokines in HS patients compared with healthy controls, while the HS patients had high proinflammatory cytokine content in the pus [24]. While macrophage content and profile did not change significantly, the profile did seem to be worsened (M1:M2 ratio) with a concomitant decrease in macrophage content, and a reported improvement in disease by the patients. This may imply that AT macrophages are actively involved in countering inflammation, rather than being inert tissue influenced by pathology elsewhere.

Overall, we see higher levels of hsCRP and IL‐6 in the patient group, indicative of systemic inflammation that support the systemic implications of HS beyond its cutaneous manifestations. Also, the neutrophil‐to‐lymphocyte ratio at 3.0 indicates increased immune activity. The ratio is similar to levels earlier reported in HS patients with Hurley Stages II and III [25, 26], but in essence, it is a mild inflammation response and thus not a key contributing factor to low‐grade inflammation. From a broader perspective, we did not observe an abnormal blood inflammation profile in HS patients when we compared it to a healthy control group. The absolute levels of hsCRP and IL‐6 are only moderately elevated and the idea that systemic inflammation is a contributing factor to the disease burden and progression is therefore still speculative from these data. This is further supported by the fact that the disease state is improved without changes in systemic inflammation, which confirms the findings of Stergianou et al. [27].

The concentration of TNF‐α in the adipose tissue is not detectable, so it is difficult to determine whether Zell et al. (1997) [17] used meaningful physiological concentrations to inhibit mitochondrial respiration with TNF‐α in their study. It is, given that AT secretes TNF‐α, possible that the TNF‐α concentration in adipose tissue is higher than the plasma concentration. Mitochondrial respiration was identical in the control and HS patient groups both before and after the intervention. Since plasma TNF‐α concentration in the patient group was low before initiation of treatment and unaltered by the treatment, any possible effects of lowering TNF‐α on mitochondrial function in AT could not be detected. Additionally, we assessed succinate sensitivity as an indicator of oxidative stress, as described by Larsen et al. [28]. Elevated sensitivity to succinate can lead to a reduction in membrane potential, consequently mitigating ROS production as a compensatory mechanism. However, we did not observe any differences between groups in succinate sensitivity, thus supporting the observed relatively minor differences in inflammatory profile. Mitochondrial function has previously been shown to be closely related to inflammation and a controlling factor for changes in inflammation [29, 30, 31]. The moderate changes we see in macrophage polarisation are not reflected in mitochondrial respiration here. It remains uncertain whether the connection between AT inflammation and mitochondrial respiration is weaker than previously described or if the alterations are simply too small to be observable.

We did not have data on adherence to the treatment but a solid increase in quality of life was observed, which serves as a good marker that the patients did follow the treatment regimen. Furthermore, despite not being statistically significantly changed, the clinical scores improved slightly, supporting the notion of good adherence to the treatment. Although we see improvements in DLQI and clinical measurements after the treatment, anti‐TNF‐α might not be the best treatment for HS, since TNF‐α is not the main driver of the disease. A new treatment regimen in HS targets IL‐17 and Th1 with promising efficacy on disease and a decrease in overall inflammation in plasma [32]. They observed no change in nonlesional skin while decreasing inflammation in lesional and perilesional skin. The relationship between inflammatory skin disease and a neighbour tissue expression of anti‐inflammatory characteristics highlights the complexity of the inflammatory response and adaptations. In this case, HS patients were not an optimal model for systemic inflammation and how improvements in systemic inflammation could affect AT. Future research in HS patients should involve both affected and nonaffected areas to further describe and understand the inflammation responses in relation to the disease.

### 4.1 Conclusion

Adalimumab improved self‐reported quality of life but with no statistically significant changes in clinical scores. A novel observation was that mitochondrial respiration in HS patients is comparable to healthy controls and not influenced by the treatment. The AT macrophage profile is more anti‐inflammatory than in healthy controls, but this did not link to systemic low‐grade inflammation markers and accordingly, adalimumab treatment did not influence this.

## Author Contributions

**Ronni Eg Sahl:** conceptualization (equal), data curation (lead), formal analysis (lead), investigation (lead), methodology (lead), project administration (lead), writing – original draft (lead). **Axel Illeris Poggi:** data curation (supporting), investigation (supporting). **Valdemar Wendelboe Nielsen:** investigation (supporting). **Yiqiu Yao:** investigation (supporting). **Ioanna Patsi:** data curation (supporting), methodology (supporting). **Steen Seier Poulsen:** data curation (supporting), methodology (supporting). **Flemming Dela:** supervision (supporting). **Steen Larsen:** data curation (supporting), investigation (supporting), methodology (supporting), supervision (supporting), writing – original draft (supporting). **Simon Francis Thomsen:** conceptualization (equal), funding acquisition (equal), project administration (supporting), supervision (supporting). **Jørn Wulff Helge:** conceptualization (equal), formal analysis (supporting), funding acquisition (equal), project administration (supporting), supervision (lead), writing – original draft (supporting).

## Disclosure

The authors have nothing to report.

## Conflicts of Interest

The authors declare no conflicts of interest.

## Data Availability

The data that support the findings of this study are available from the corresponding author upon reasonable request.
